# The Fewer Reasons, the More You Like It! How Decision-Making Heuristics of Image Quality Estimation Exploit the Content of Subjective Experience

**DOI:** 10.3389/fpsyg.2022.867874

**Published:** 2022-06-21

**Authors:** Tuomas Leisti, Mikko Vaahteranoksa, Jean-Luc Olives, Veli-Tapani Peltoketo, Jukka Häkkinen

**Affiliations:** ^1^Department of Psychology and Logopedics, Faculty of Medicine, University of Helsinki, Helsinki, Finland; ^2^Huawei Technologies Oy (Finland) Co., Ltd., Helsinki, Finland

**Keywords:** image quality, judgment and decision-making, heuristics, attention, subjective experience, image quality attributes

## Abstract

Imaging science has approached subjective image quality (IQ) as a perceptual phenomenon, with an emphasis on thresholds of defects. The paradigmatic design of subjective IQ estimation, the two-alternative forced-choice (2AFC) method, however, requires viewers to make decisions. We investigated decision strategies in three experiments both by asking the research participants to give reasons for their decisions and by examining the decision times. We found that typical for larger quality differences is a smaller set of subjective attributes, resulting from convergent attention toward the most salient attribute, leading to faster decisions and better accuracy. Smaller differences are characterized by divergent attention toward different attributes and an emphasis on preferential attributes instead of defects. In larger differences, attributes have sigmoidal relationships between their visibility and their occurrence in explanations. For other attributes, this relationship is more random. We also examined decision times in different attribute configurations to clarify the heuristics of IQ estimation, and we distinguished a top-down-oriented Take-the-Best heuristic and a bottom-up visual salience-based heuristic. In all experiments, heuristic one-reason decision-making endured as a prevailing strategy independent of quality difference or task.

## Introduction

It is deceptively easy to regard the visual quality of an image as something essentially objective. An image can be described almost exhaustively by measuring the light emitted from a display or reflected from a print. Therefore, it also appears that quality can be measured solely using this information. Nevertheless, only subjective evaluations offer first-hand data about quality, and even instrumental measurements of quality require a subjective reference, which the measurements eventually try to predict (Engeldrum, 2004b).

Why do subjective estimations play such a significant role if images are fully described by objective measures? The answer can be sought from the general idea of quality as a property, which can order images according to their utility, excellence, beauty, or simply preference (Janssen and Blommaert, 1997; Keelan, 2002; Engeldrum, 2004a). This ordering is logically possible only if quality is defined as a one-dimensional quantitative property. Hence, there must be a rule for how the inherent multidimensionality of image can be transformed into this quality dimension. The result, a quality scale, should be able to put products in order and should therefore conform both to the axioms of transitivity and completeness and to the general opinion about the quality in order to correctly predict consumer choices, which usually is the final goal of marketers, engineers, and designers. The quality scale is typically operationalized as a mean opinion score (MOS), which simply is a mean quality rating of the image given by a group of participants in subjective image quality (IQ) tests.

To understand the relation between the physical properties of an image and its quality scores, which reflect how these properties are perceived, much of the methodology of subjective IQ estimation has been adopted from psychophysics (Keelan, 2002; Jin et al., 2017). Imaging science has thus conceptualized quality mainly as a physical, objective phenomenon, and its psychological counterpart is only a subjective reflection of objective quality. The ultimate goal is to find appropriate psychophysical functions that can predict subjective ratings from objective properties of the image (Keelan, 2002; Engeldrum, 2004b; Jin et al., 2017). This is feasible, of course, if only one dimension, such as blur, varies between the images. However, when IQ is multidimensional, that is, images vary simultaneously according to several dimensions, such as blur, noise, contrast, and color saturation, the situation is more complicated because only some visual dimensions, such as color saturation, lightness, and hue, are integrated at the perceptual level (Garner and Felfoldy, 1970). Most quality dimensions are perceived as separate, and therefore, the multidimensional quality estimation task is essentially a judgment and decision-making problem. This is evident, for example, when the viewer must decide between blurry and noisy images. The first aim of this paper is to examine how research participants recruited to IQ tests make decisions about quality and how these decisions influence the test results.

Another challenge for the traditional psychophysical approach is that, psychologically, estimation of IQ is also a result of active, experiential, and interpretative activity. For example, we tend to think blur is an IQ defect, but professional photographers also use blur as an artistic effect or as a way to attract viewers’ attention. Furthermore, new camera phones use “bokeh” effect to create artistic-looking photos, simulating the narrow depth of field typical for photographs taken with professional cameras. Whether the viewer considers blur an advantage or a disadvantage therefore depends on the *interpretation*; participants tend to give higher ratings to IQ when blur is considered artistic (Radun et al., 2008). Thus, subjective quality is not merely a psychophysical function of physical image properties; quality estimations result from both subjective interpretation and objective, perceptible features of the image.

“Artistic” is one IQ attribute that is difficult to define using objective properties of an image, but it is not the only one. When people are asked to describe the reasons for their IQ ratings, they can use similar, rather abstract attributes such as “warm,” “atmospheric,” “good colors,” “vivid,” “soft,” or “fresh” (Leisti et al., 2009; Radun et al., 2016; Virtanen et al., 2019, 2020). Lower level properties, such as sharpness, noise, contrast, or color fidelity, are easier to measure objectively, but they do not present an exhaustive description of the subjective factors that determine the viewer’s experience of IQ (Radun et al., 2016). A wide semantic gap exists between the subjective descriptions of quality and the objective properties of an image. Therefore, the second aim of this paper is to examine what information research participants use in their decisions; we are interested in the *decision space* (Nyman et al., 2010) from which the attributes used in the quality evaluation are sought.

### Interpretation-Based Quality: Probing the Experience of Quality

When quality evaluation is based on subjective aspects that are nearly impossible to measure objectively, the question is how to gain information about the crucial quality attributes and build a model describing the associations between physical properties, these subjective quality attributes, and overall quality. The *Interpretation-Based Quality* (IBQ) method was developed to understand the experiences that people exploit in their judgments of IQ (Nyman et al., 2005, 2006; Radun et al., 2008, 2010; Virtanen et al., 2019, 2020). Initially, the purpose was to bridge the semantic gap between low-level properties of the image and high-level attributes of subjective experience by examining how viewers interpret differences in quality in natural images. Radun and colleagues (Radun et al., 2008, 2010; Virtanen et al., 2020) did this by gathering subjective descriptions of quality from interviews of research participants about the relevant aspects of their quality judgments, analyzing these descriptions qualitatively, and exploring the underlying structure and dimensionality between these descriptions and physical stimuli. The IBQ method incorporated these interviews into experimental designs and controlled laboratory conditions such that the data provided by descriptions could be associated with instrumental data and experiment parameters using statistical and computational methods (Radun et al., 2008, 2010; Eerola et al., 2011).

The approach employed by the IBQ approach therefore represents *subjective-to-objective* mapping, which first describes the subjective phenomena, such as the subjective experience of IQ as it manifests in quality descriptions in this case, and then seeks the objective counterparts of the subjective attributes of experience (see Albertazzi, 2013; Felin et al., 2017). A similar approach has been employed in vision science when, for instance, visual illusions are used to study the functioning of the human vision system (Albertazzi, 2013). After describing the relevant dimensions of experience, models can be created that predict the quality experience and subsequent ratings on the basis of objectively measurable physical metrics (e.g., Eerola et al., 2011). This kind of top-down, interpretative approach complements the prevalent *objective-to-subjective* mapping tradition in IQ, adopted from psychophysics.

A significant difference exists between the subjective-to-objective and the objective-to-subjective approaches. When IQ estimation is considered similar to the estimation of lightness or contrast in simple stimuli, any disagreement between participants is regarded as error. If subjective experience is considered primary, however, quality evaluation is a preference task and no objectively correct answer exists. This preferential aspect is evident in the case of no-reference IQ in particular, where no “original,” unprocessed reference image exists, only different versions of the same scene (Engeldrum, 2004a). Photographs may not have, for example, a correct solution for lightness levels or color balance; instead, many equally natural solutions can exist (Felin et al., 2017). Moreover, it is questionable whether consumers want realistic photographs because they seem to prefer more colorful images (Janssen and Blommaert, 1997). Although the objective-to-subjective approach works well when estimating the visibility, thresholds, and saliency of image artifacts, it does not capture the meaning of these artifacts to the participant, particularly in complex, multidimensional everyday environments. Preferential or esthetic attributes, such as contrast, naturalness, and colorfulness, cause even more difficulties because their effects on IQ cannot be determined by visibility (Keelan, 2002).

### What Is the Subjective Experience of Quality and Why Is It Important?

As the IBQ approach claims to examine the *subjective experience* of quality, it should also define this phenomenon. In philosophy and psychology, subjective experience refers to the pure, non-reflective content of consciousness such as seeing red or feeling anger or pain (Morsella, 2005; Baumeister and Masicampo, 2010). All of the relevant low-level phenomena of quality, such as blur, grain, colors, contrast, and lightness level, are experienced somehow and the participants’ ratings reflect judgments based on these experiences.

How are the physical properties of images experienced? The human visual system (HVS) consists of numerous feature detectors that are sensitive to different aspects of the visual scene such as line orientations, spatial frequencies, movement, and color (Zeki and Bartels, 1999; Kravitz et al., 2013). These functional aspects of the HVS have been adopted to the IQ metrics decades ago (Teo and Heeger, 1994; Sheikh and Bovik, 2006); the HVS-based IQ models use similar channels to process image information and apply knowledge about HVS properties, such as contrast sensitivity, to estimate the visibility of the defect. The problem with these HVS models lies in the subsequent step: what to do with these HVS-adapted visual features? The usual solution is just to sum all types of degradations, using the Minkowski metric rule, to derive an overall estimation of IQ (Engeldrum, 2002; Keelan, 2002; Jin et al., 2017). This bottom-up approach does not, however, take into account the meaning of the visual information. HVS-based models have been criticized for not considering, for instance, the structure of the image, leading to low correlations with MOS values (Wang et al., 2004).

What kind of picture emerges if the problem is approached from top-down and IQ is conceptualized as a subjective experience? In cognitive neuroscience, there is a converging consensus that the role of subjective experience is to integrate information from massive parallel sets of independent processors in the brain (Dennett, 2001; Baars, 2005; Morsella, 2005; Dehaene et al., 2006; Morsella et al., 2016). Therefore, the results from the detectors in the HVS are not experienced as such, and more importantly, their information is not mechanically summed in order to achieve an estimation of IQ. Instead, subjective visual experience is a result of active interaction between the bottom-up and top-down processes and interpretation of the resulting information, based on current task needs (O’Regan and Noë, 2001; Hochstein and Ahissar, 2002; Lappin, 2013). Visual experience emerges, when the bottom-up or feed-forward processes first provide a gist of the visual scene, and the top-down processes then amplify the details by focusing attention on the task-relevant aspects (Hochstein and Ahissar, 2002; Crick and Koch, 2003; Lamme, 2006; Kravitz et al., 2013). The “bandwidth” of subjective experience is relatively narrow, thus, only a minor subset of all visual information is represented in detail (Cohen et al., 2016). Eye movements, guided by involuntary and voluntary attention, are needed to acquire details over the entire visual scene.

Information from the feature detectors is integrated into percepts that are relevant from the action point of view (Cisek and Kalaska, 2010; Morsella et al., 2016). For example, when a participant’s task is to evaluate the IQ, information about different IQ features becomes available in subjective experience, enabling the individual to make the required decisions and complete the task. The interpretation of the task and image properties has a significant effect on the attention regulation of the participant in a quality estimation task (Radun et al., 2016). Attentional focus amplifies and attenuates visual information at the visual cortex, changing the way the image and its quality is experienced (Hochstein and Ahissar, 2002; Dehaene et al., 2006; Tse et al., 2013). What people experience is therefore highly context-dependent. Perception, decision-making, and motor control form a tightly interconnected, dynamic system (Cisek and Kalaska, 2010).

### How Subjective Experience Becomes a Pairwise Choice

The two-alternative forced-choice (2AFC) method is the basis of many IQ grading systems (Keelan, 2002; Keelan and Urabe, 2003). The 2AFC method is sensitive and it enables testers to describe quality differences in just noticeable differences (JNDs; Keelan, 2002). Unlike category scales, such as Likert, JND provides an unambiguous, well-defined measure of quality difference between two images. It is therefore the unit of measurement of IQ standards such as quality ruler (Keelan and Urabe, 2003; Jin and Keelan, 2010).

The drawback of the 2AFC method is a narrow dynamic range. Large amounts of blur, noise, or color distortion exceed the threshold for consciousness without intention. Detection of artifacts is not probabilistic at this stage, and IQ cannot be scaled using probabilistic methods. Even when differences are multidimensional, saliency of certain attributes captures involuntary attention, providing a heuristic reason for rejecting the photograph. These kinds of decision tasks thus rely on simple choice heuristics, require little voluntary search for defects, and are easy, fast, and reliable (Gigerenzer et al., 1999). There is not much practical difference whether large differences in the task are one-dimensional (supra-threshold task) or multidimensional (heuristic task). Table 1 provides a schematic categorization of different IQ estimation tasks, differentiating them by two factors, dimensionality and quality difference within an image pair.

**Table 1:** Image quality estimation tasks categorized according to the dimensionality of the differences and the magnitude of the overall quality difference.

	Small quality difference	Large quality difference
One-dimensional differences	Threshold task	Supra-threshold task
Multidimensional differences	Conflict resolution task	Heuristic task

When quality differences between alternatives within a pair are small, less than two JNDs, one-dimensional and multidimensional tasks become completely different tasks. I will call them threshold tasks and conflict-resolution tasks. When JND less than two in a one-dimensional task is caused solely by a small difference in visibility of a single attribute, in the multidimensional task, it can also be caused by conflict between dimensions. How participants make judgments and choices between blurry and noisy images, for instance, should be very different from one-dimensional threshold tasks, typical for psychophysics, where comparisons are made between images with different levels of blur.

When conflict emerges, a voluntary decision about the importance of different attributes is required. Here, a deliberative approach is an automatic brain reaction (Alter et al., 2007; Botvinick, 2007), which involves more detailed analysis of the attributes and conscious reasoning about the importance of different attributes (Shafir et al., 1993) in order to resolve the conflict. Subsequently, the decision process slows down because the task requires serial top-down control. Attributes in subjective experience form the “decision space” (Nyman et al., 2010; Morsella et al., 2016), which represents the aspects governing the choice.

Research on judgment and decision-making has traditionally suggested that a normative solution to such multidimensional choice problems is a compensatory strategy, which uses all available data and weights it according to its importance (e.g., Payne et al., 1988). In most cases, compensatory strategy requires too much time and cognitive resources (Simon, 1955), and there is much evidence that heuristic, one-reason strategies perform well in most real-life choices (Gigerenzer et al., 1999). In other words, for most decisions, only one reason is required for a satisfactory choice, which diminishes the time and effort involved. Some studies suggest that decision strategies gradually shift toward a more heuristic style, and compensatory strategies are more typical for novices (Garcia-Retamero and Dhami, 2009; Leisti and Häkkinen, 2018). Experts can therefore rely on more efficient strategies in their decisions.

When there is only one reason, the question that follows is what determines the specific reason. So far, it is known that the attributes unfolding in the decision space are dependent on the context such as image content (Radun et al., 2008). Not only is the visibility of artifacts dependent on the content, but also personal interpretation of image properties differs between contents. Therefore, solving the decision problem returns to the question of how the alternatives are interpreted and experienced. Subjective phenomena always have personal meaning that is not contained within the physical stimuli (Albertazzi, 2013), thus, the view that IQ consists of static component attributes, or “-nesses” that are subjective representations of objective image properties (Engeldrum, 2004b) becomes problematic.

The IBQ approach is based on the attribute data that participants produce spontaneously as reasons for choices. This differs from typical approaches that rely on psychophysical threshold tasks or category scales, where experimenters specifically prompt observers to evaluate quality on predefined attributes scales. The weakness of these ready-made scales is that the subjective decision space of the participants cannot be known beforehand, as the emerging set of attributes is dependent on the personal interpretation of the task (Radun et al., 2016). Asking consumers to evaluate products with a predefined attribute may interfere with their personal approach by diverting attention away from attributes that they would normally consider important (Tordesillas and Chaiken, 1999; Radun et al., 2016). This may not only change the weighting of the individual attributes (Wilson and Schooler, 1991) but may also interfere with the consumer’s experience of quality, which is dependent on the aspect receiving attention (Tse et al., 2013; Yamada et al., 2014).

### Purpose of the Study

The purpose of this study is to describe the decision space that unfolds to participants when they are required to make decisions in a 2AFC task and how they use the attributes that emerge in this space. We are specifically interested in small multidimensional differences present between flagship camera phones. This is a context where we expect the quality deviations to be most dependent on experiential aspects and personal taste instead of defects, for which there are several instrumental measures available. Our approach is exploratory and focuses on the following aspects: IQ differences within image pairs, numbers of reported reasons, decision times, and specific IQ attributes.

Our introduction opened up two orthogonal research questions, the first concerning the roles of subjective experience and decision-making in IQ estimation, and the second differences between small and large quality differences. Cross-sections of these research questions yield four specific themes: what kind of reasons is reported when differences between images are small or large and what kind of decision strategies are applied to the attributes that emerge from those experiences when differences are small or large?

## Experiment 1

### Methods

#### Participants

Participants (*N* = 32) were recruited from the student email lists of the University of Helsinki to participate in an experiment about decision-making and IQ. The number of participants was dictated by the counterbalancing of the 32 stimulus images evenly in each condition between participants. We tested participants for visual acuity (Lea numbers), near contrast vision (near F.A.C.T.), and color vision (Farnsworth D15). All participants passed the tests. They received a movie ticket as compensation for their participation. The mean age of the participant group was 26.1 years (*SD* = 4.4). Of participants, 27 were females and five males.

#### Stimuli

**Figure 1:**
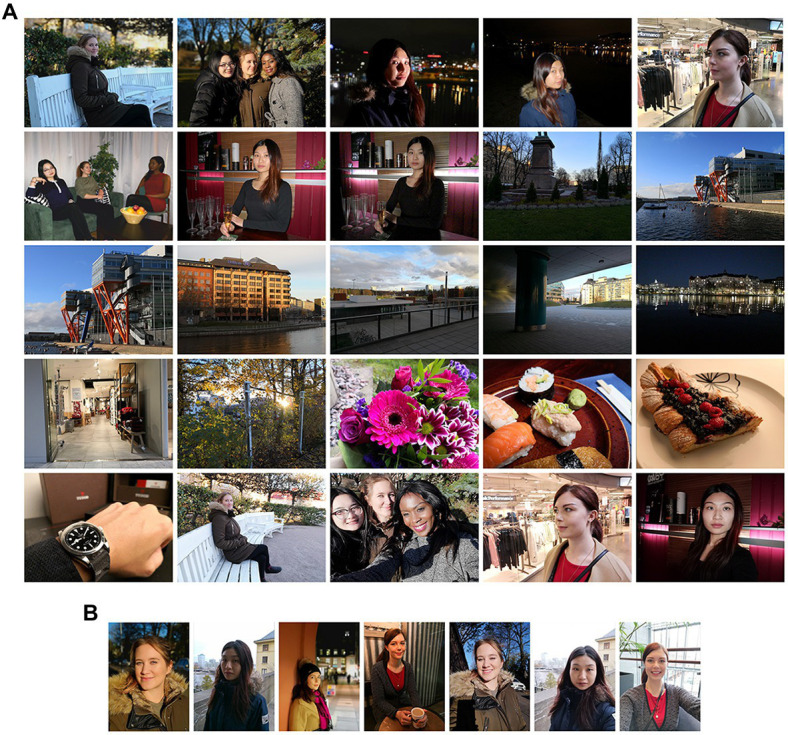
Image contents used in the study. Of 32 contents, 25 were in landscape mode **(A)** and seven in portrait mode **(B)**. For certain contents, there were duplicates taken with back and front camera or with different zooming or flash settings. The models gave a written consent for the publication of the photographs.

The stimuli were based on 32 predetermined image contents that represented typical use scenarios of camera phones (Figure 1). Scenarios were selected according to their location in photospace (Keelan, 2002), which is a frequency distribution of photographs taken by ordinary users of point-and-shoot cameras, located in two dimensions according to their shooting distance and illumination level. Most frequent use cases in photospace were stressed in content selection, but the selection also included multiple skin colors and challenging cases, defined by the experts (authors MV and J-LO).

We used four flagship camera phones from leading manufacturers to create four different versions for each content. A professional photographer took five different photographs of each content with each device. From these five photographs, the photographer chose the best image for the experiment based on his own opinion. The images (*N* = 128) were rescaled to 2,560 × 1,440 (landscape) or 1,080 × 1,440 (portrait) resolution. We presented each image on Eizo 27” ColorEdge CG2730 display, calibrated to sRGB color space, 120 cd/m^2^ luminance, 2.2 gamma, and D65 white point. The displays had no known differences in uniformity. The ambient illumination in the laboratory was set at 20 lux, using D65 fluorescent lamps.

The stimulus material is available upon request from the corresponding author.

#### Procedure

After providing informed consent and passing the vision tests, participants were given the instruction that the task would be a paired comparison task, where they should choose the better of the two images. We emphasized that the task is subjective, i.e., there are no right or wrong answers. We asked the participants to consider which of the images they would save for general use, e.g., putting it in a photo album or on social media or showing it to family or friends.

We used the PsychoPy (Peirce, 2007) environment for creating the experiments. We used the 2AFC method, thus, with 32 contents and four devices for each content, there were altogether 192 image pairs for the 2AFC task. We divided the experiment into 32 blocks; in each block, participants evaluated six image pairs of single content in random order. In half of the blocks, we employed the IBQ method (Radun et al., 2008); participants provided explanations for their decisions. In the rest of the blocks, the participants only made choices, without explanation, to reduce the experiment duration.

The conditions with or without explanations formed super-blocks, consisting of half of the contents. We varied the order of these super-blocks and counterbalanced them between participants. In other words, half of the participants completed all of the silent contents first and then the contents with explanations, and vice versa. We counterbalanced also the contents within the super-blocks and randomized them between participants.

Within each trial, two stimulus images were presented on two parallel calibrated displays at the same time, and a third non-calibrated display next to the keyboard was used for answering. Simultaneously with the images, two buttons appeared on the response display for participants to indicate their preference. After selecting the better image, the text field appeared below the buttons for the participant to explain their choice in Finnish (in explanations condition). After this, the participant proceeded to the next image by pressing the “next” button below the text field. Participants could not proceed if they had not indicated a choice or the text field was empty. Between trials, a neutral gray rectangle replaced the images for 500 ms.

The Ethics Review Board in Humanities and Social and Behavioral Sciences of the University of Helsinki approved the experimental protocols of this study (decision no. 40/2017).

#### Data Analysis

##### Quantitative Analysis

When the images produced by the four different devices were compared pairwise, the total number of choices was six for each content. With 32 image contents, the total number of pairs was 192. We transformed the choice probabilities in each pair further into just noticeable differences (JNDs). We first used the logit transformation:


(1) $$\mathrm{logit}\;\left(p\right)=\ln\left(\frac{p}{1-p}\right)$$

where *p* and 1-*p* represent the choice probabilities of the images. We then rescaled logit values into JND values using the formula


(2) $$JND=\frac{\mathrm{logit}\left(p\right)}{\mathrm{logit}\left(.75\right)}$$

##### Qualitative Analysis

In the qualitative analysis of explanations for the choices, we followed the approach employed by Radun et al. (2008, 2010). We used Atlas.ti software (Muhr, 2004) for this purpose. Before the analysis, we imported the explanation data with identifiers for participant ID, image content, experiment trial, and image such that each attribute could be linked to each trial, stimulus, and participant. In the first phase, we coded each explanation with attribute codes that we found in the explanation, without making any interpretation of the meanings of the attributes; e.g., the explanation, such as “This photo is blurry and faded,” was coded with codes “blurry” and “faded.” In other words, codes denote a certain attribute that has been used to explain the selection or rejection of a certain image from a certain pair. We made the coding blindly, without knowing the identities of the cameras and the contents. In the second phase, we further streamlined the coding scheme by creating more exact definitions for each attribute code and merging similar attributes (e.g., “blurry,” “unsharp,” and “not sharp” into “unsharp”). After this, we re-analyzed the explanation data using these definitions and corrected possible deviations from these definitions.

##### Quantitative Analysis: Attributes

In addition to counts, we calculated other descriptive measures for attributes. First, we calculated measure of *accuracy* for each attribute *i* in each pair *j*:


(3) $$\mathrm{accura}cy_{ij}=|\frac{n_{p}-n_{q}}{n_{p}+n_{q}}|$$

where *n*_p_ and *n*_q_ are the counts of the attribute in each image *p* and *q*. When accuracy measures were calculated over several pairs or attributes, means were weighted according to the count of the attributes. We then calculated a measure of valence for each attribute to determine whether the attribute was considered positive or negative. This measure describes the proportion of attribute mentioned with the selected or rejected image in relation to all occurrences:


(4) $$\mathrm{valenc}e_{ij}=|\frac{n_{\mathrm{selected}}-n_{\mathrm{rejected}}}{n_{\mathrm{selected}}+n_{\mathrm{rejected}}}|$$

where *n*_selected_ are *n*_rejected_ counts of attribute *i* for selected and rejected images in each pair *j*. Overall valence was calculated as the weighted mean over all pairs, and the weight was determined by the attribute counts.

### Results and Discussion

#### Subjective Attributes

Our qualitative analysis yielded 52 subjective IQ attributes^1^ that participants mentioned more than once (Appendix A). Participants mentioned some aspect of sharpness, color, or lightness level most often as the principal reason for choice. In addition, there were 30 positive and 21 negative attributes that occurred only once in explanations and could not be merged with other attributes; these were omitted from further analyses.

#### The Number of the Reported Reasons in the 2AFC Trials

From the viewpoint of the reported reasons, participants’ choices can be explained by a rather simple heuristic strategy: in most trials, participants reported one attribute (Mean = 1.2; Standard Deviation = 0.51; later abbreviated as *M* and *SD*, respectively) for selecting and one attribute (*M* = 0.9; *SD* = 0.62) for rejecting an alternative. Based on the valence calculated for each attribute, only a small minority of the attributes given to the selected alternative were negative (*M* = 0.06; *SD* = 0.27). The same applied to the positive attributes given to the rejected alternative (*M* = 0.8; *SD* = 0.28). The number of attributes was approximately the same for all contents; the maximum mean number of positive attributes for selecting was 1.3 and the minimum 0.9. The corresponding figures for rejection and negative attributes were 1.1 and 0.5. This kind of answering scheme may have been also prompted by the test design, which included one field for explaining the selection and another for explaining the rejection.

#### The IQ Attributes and the Magnitude of the Quality Difference

**Figure 2:**
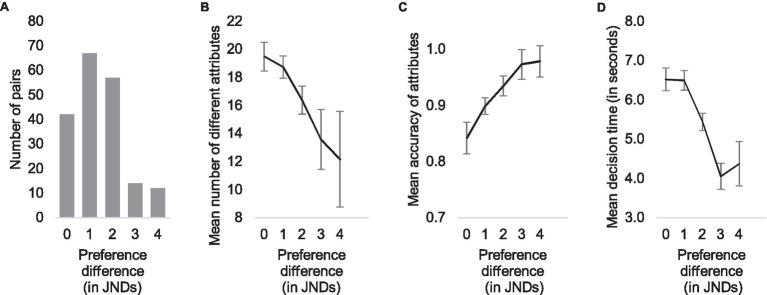
Distribution of quality differences **(A)** and the qualitative shift between one and three just noticeable differences (JNDs) illustrated by three measures: mean number of different attributes **(B)**, mean accuracy of attributes **(C)**, and mean decision time **(D)**.

We transformed the choice distributions within the pairs to JND values using logit transformation and then divided all 192 pairs into groups according to the quality differences between the alternatives. The step between groups was one JND. The first group (JND = 0) consisted of all pairs with a difference below 0.5 JNDs, the second group (JND = 1) with a difference between 0.5 JND and 1.5, etc. Figure 2A shows the distribution of pairs in these quality difference groups.

After dividing the trials into categories according to their quality differences, we calculated the mean number of different attributes, the mean accuracy of the attributes, and the mean response time in each category and plotted the results in Figure 2. Visual examination of Figure 2 suggests that decisions in pairs with large differences are made with a smaller number of different attributes (Figure 2B), with high accuracy (Figure 2C) and rapidly (Figure 2D), whereas larger variety of attributes, lower accuracy, and slower decisions are typical for small differences. Correlational analysis supports this impression: spearman correlations between quality difference (in JNDs) and the number of different attributes, mean accuracy, and mean decision time were *r* (190) = −0.49, *r* (190) = 0.58, and *r* (190) = −0.60, respectively (all *p* < 0.001).

**Figure 3:**
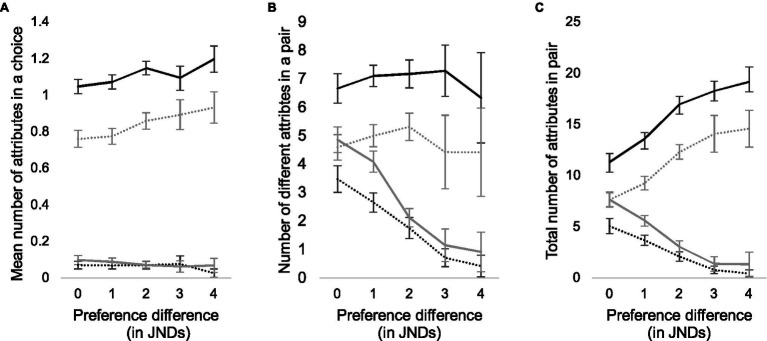
Mean number of attributes that participants used in each pair according to their quality difference **(A)**, number of different attributes that participants used **(B)**, and total number of reasons given in each pair **(C)**. Black lines represent the number of chosen alternatives, gray lines the number of rejected alternatives, and solid lines and dashed lines the positive and negative attributes, respectively.

We further analyzed the total number of attributes in each pair given by all participants, the total number of *different* attributes given by all participants, and the mean number of attributes given in each trial, and divided the attributes according to their valence (positive or negative) and whether they were given to the selected or the rejected alternative. The decrease in the number of different attributes (Figure 2B) is mainly due to a decrease in the number of reasons that conflict with the majority choice (Figure 3B). In other words, when the difference between the images is small, both the number of positive attributes for the rejected alternative and the number of negative attributes for the selected alternative are larger (Pearson correlation coefficients in Table 2, second row).

**Table 2:** Spearman correlation coefficients between quality difference and total number of attributes, mean number of different attributes, and mean number of attributes in a choice.

	Selected	Rejected
	Positive	Negative	Positive	Negative
Number of attributes	0.75^***^	−0.65^***^	−0.75^***^	0.65^***^
Mean number of different attributes	0.05	−0.6^***^	−0.73^***^	0.03
Mean number attributes in a choice	0.3^***^	−0.07	−0.21^**^	0.27^***^

The positive and negative attributes are separated both for the selected and the rejected alternative.

*** *p* < 0.001;

** *p* < 0.01.

When participants and pairs are examined individually, the number of reasons increases slightly as the preference difference increases (Spearman *r* = 0.28; *p* < 0.001; Figure 3A). This is due to the increasing numbers of positive attributes for the selected alternative and negative attributes for the rejected alternative (Table 2, third row). These increases are, however, rather low: an average from 1.05 to 1.2 for positive attributes and from 0.76 to 0.93 for negative attributes. Finally, Figure 3C illustrates the total counts of positive and negative attributes for better and worse alternatives as a function of quality difference (also Table 2, first row).

On an individual level, only one reason is usually required to justify a choice, independent of quality difference. However, when we examine the number of different attributes over a larger group of participants, the decision space expands significantly when differences are small. A larger number of different attributes indicates that with small quality differences participants’ attention diverges to different image properties and image areas due to a lack of salient quality defects. However, participants’ prevailing decision strategy does not seem to change at different quality levels.

**Figure 4:**
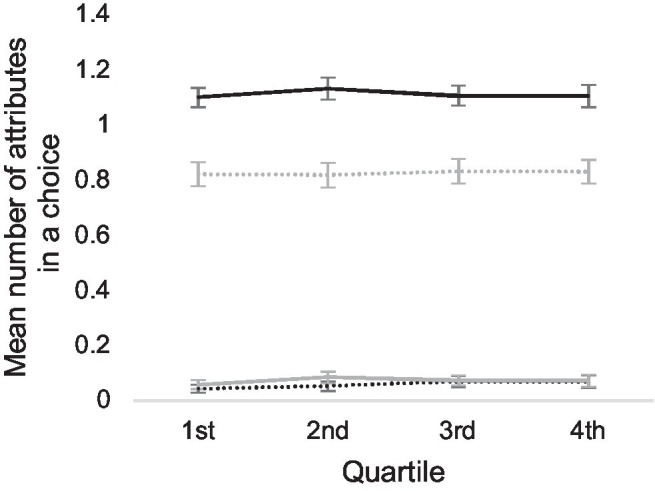
Mean number of attributes in all choices, divided into quartiles according to the decision times. The *x*-axis represents the different quartiles of decision times. Black lines represent the number of chosen alternatives, gray lines the number of rejected alternatives, and solid lines and dashed lines the positive and negative attributes, respectively.

To test the hypothesis that participants use the same decision strategy in all of their choices, independent of quality level or other factors, we divided all 3,074 choices, where reasons for choices were given, into quartiles according to their decision times and calculated mean numbers of attributes given in each quartile. The result is shown in Figure 4, suggesting that no radical change in decision strategy occurs when participants use more or less time to make a choice. We tested this by estimating the coefficient *B*_1_ in a linear regression model:


(5) $$y=B_{0}+B_{1}t+B_{2}id_{1}+B_{3}id_{2}+B_{4}id_{3}\ldots +B_{n+1}id_{n}$$

where *t* indicates the decision time and *y* the number of the attributes. To control for the effect of individual differences, we included participant identities in the model as dummy variables *id*_1_…*id*_n_ that indicated the identity of the participant 1…n with value of 1, the value being otherwise 0.

According to the estimated coefficient *B*_1_ of all models, longer decision times do not mean a larger number of attributes. On the contrary, longer decision times are related to a smaller number of positive attributes for the selected alternative, the value of the coefficient *B*_1_ being −0.0052 (the standardized coefficient *β*_1_ was −0.49), suggesting that longer decision times are due to participants having difficulties in finding a reason to make a choice. Student’s *t*-test showed that the coefficient *B*_1_ differed from zero [*t*(3,071) = −2.63; *p* = 0.009]. The proportion of the variance *R*^2^ explained by the model was 0.11.

The model predicting the number of negative attributes for the selected alternative indicated that there is a slight increase in the number of attributes with increasing decision time, as the coefficient *B*_1_ was 0.002 [*β*_1_ = 0.042; *t*(3,071) = 2.17; *p* = 0.03; *R*^2^ = 0.05]. This implies that longer decision times may involve a conflict that the negative aspects of the selected alternative induce to the choice.

Nevertheless, longer decision times do not mean an increase in the number of positive or negative attributes given to the rejected alternative, as the *B*_1_ coefficient did not differ from zero in those models, according to *t*-test [*t*(3,071) = 0.190; *p* = 0.85 and *t*(3,071) = −1.84; *p* = 0.066, respectively].

Only the number of attributes that participants use to explain selection, not rejection, are related to decision times, suggesting that heuristic strategy prevails at all time spans. In this strategy, participants seek reasons for selecting certain alternative and may hesitate if the preferred alternative has also negative, conflicting properties. Conflict between positive attributes of both alternatives in a pair, however, does not cause an increase in decision times. Participants appear to focus on finding plausible reasons for selecting one alternative and do not use additional time to deliberate over the positive aspects of both alternatives, indicating a form of confirmation bias.

#### The Use of Attributes When Quality Differences Are Large and Small

We further analyzed how different subjective attributes are used in pairs with large and small quality differences. We used two JNDs as a cut-point and divided the pairs into two categories: the small difference category (difference less than two JNDs) and the large difference category (difference more than two JNDs). We then calculated the proportion of each attribute in these small and large difference categories.

We based our analysis on the hypothesis that the use of attributes can contribute to small quality differences through three mechanisms: (1) divergent attention to several attributes and dilution of overall difference; (2) lower accuracy of attributes, caused by smaller visual differences; and (3) use of attributes with ambiguous or low diagnostic value due to lack of more diagnostic attributes.

**Figure 5:**
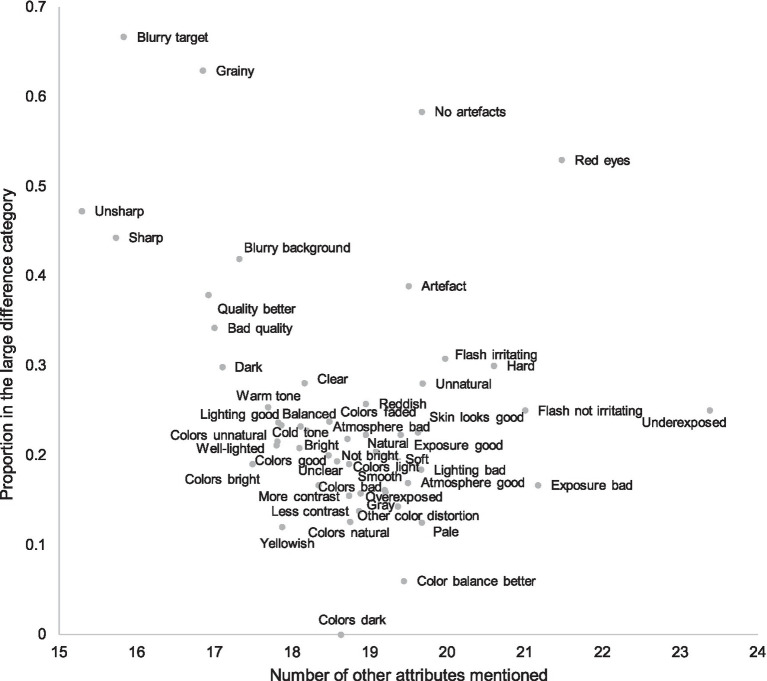
Proportion of attributes in the large difference category and number of other attributes mentioned in the pair. The subjective attributes most often mentioned for large quality differences were less often accompanied by other attributes. However, when quality differences were small, there was a plethora of different subjective attributes, most referring to color balance and general lightness level. In other words, choices in large quality difference pairs is usually explained by smaller number of subjective attributes than in small quality difference pairs.

In Figure 5, we plotted the proportion of the attributes in the large difference category against the number of all other attributes mentioned within the pair. For instance, if the attribute in question is “sharp,” we counted all other attributes used by all participants, such as “grainy” and “natural colors.” The figure illustrates that most subjective attributes appear to describe rather small differences, and large differences are associated with a few clear defects, such as blur, noise, or red eyes. Hence, when differences are smaller, participants’ attention toward different attributes diverges, increasing the number of attributes. Attributes referring to colors, contrast, and lightness level are typical for the smaller differences. When quality differences are larger, salient attributes attract the attention of most participants, leading to a higher consensus and a smaller number of attributes. This is in line with our third hypothesis. In other words, people have fairly high tolerance for differences in preferential attributes and appear to focus on them only when no visible defects or artifacts exist. Detection of defects is a heuristic decision rule for the participants; in pairs where differences are large, participants make fast choices using a limited set of attributes, which clearly differentiate the alternatives.

**Figure 6:**
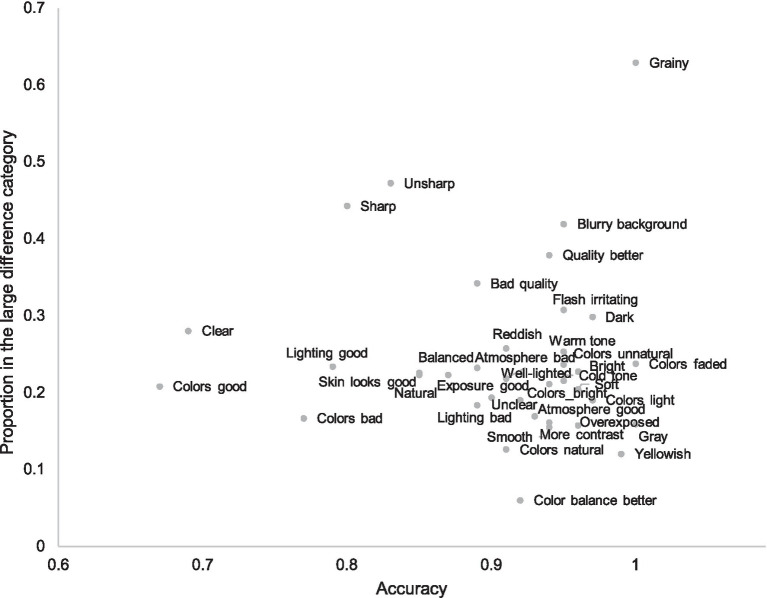
Subjective attributes according to their accuracy and their proportion in the large difference category. Only attributes with frequency more than 31 are included; the accuracy estimation of rarer attributes is biased.

Figure 6 illustrates the relation between the proportion of the attribute in the large difference category and its accuracy. It is evident that the least accurate attributes are less specific and given in pairs where quality difference is small. Such attributes are, for instance, “colors good,” “colors bad,” “lightning good,” and “clear.” However, attributes referring to sharpness are also relatively inaccurate despite their frequency in larger quality differences and apparent clear meaning. Because the attributes are brought up spontaneously, it is peculiar that people use attributes like “sharpness” when no clear, shared understanding about the sharpness difference exists. We examine this further in Experiment 2.

There are also attributes in the small difference group that are accurate, for instance, the more specific attributes referring to colors such as “colors faded,” “gray,” and “yellowish.” Figure 6 shows that weaker accuracy in smaller quality pairs is mostly caused by the use of less accurate attributes, not weaker general accuracy of all attributes. A notable exception to this rule is “sharpness.”

**Figure 7:**
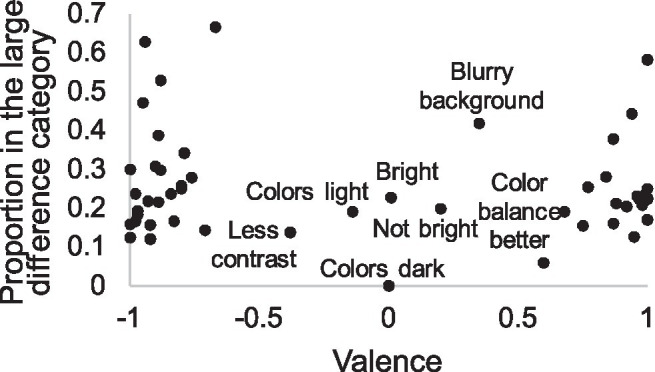
Subjective attributes according to their valence and proportion in the large difference category (for calculation of valence, see section “Data Analysis”). Most attributes align to either end of the valence dimensions. The named attributes in the middle of the valence dimension represent the small minority of all attributes, are typically mentioned in pairs with small difference, and have rather neutral meanings. Location of other attributes in these two dimensions is reported in Appendix A.

In addition to accuracy and divergent attention, the use of attributes with low diagnostic value may lead to small quality differences. For instance, the attribute “bright” does not clearly indicate whether the image is good or not and is not therefore very diagnostic, unlike the attribute “grainy,” which immediately reveals that the IQ is not good. In Figure 7, we have plotted attributes according to their proportion in large difference pairs against the valence of these attributes, showing that some attributes, typically used in small difference decisions, are neither positive nor negative, such as light colors, brightness, and blurry background. Most of the attributes, however, are unambiguously positive or negative, even when quality differences are small. Thus, heuristic quality estimation strategy seems to avoid attributes that have unclear valence and seeks plausible, justifiable reasons.

#### Difference Between Trials With Explanations and “Silent” Trials

We have earlier shown that performance in IQ estimation tasks differs slightly between trials where participants are required to give reasons for their decisions and trials, which do not have such requirement. Most importantly, participants are typically more consistent in their decisions when explanations are required (Leisti and Häkkinen, 2016, 2018). This is probably due to a more thorough information search, which also leads to more pronounced differences between alternatives (Leisti et al., 2014). We found this preference polarization also in this study; the mean preference difference was 1.65 JNDs when explanations were required and 1.46 JNDs in silent trials, suggesting that participants were more unanimous in the former condition. Despite the differences in consistency, Spearman correlation coefficient between conditions was *r*(190) = 0.89. We come back to this issue in Experiment 3.

## Experiment 2: How Threshold Task Differs From Quality Estimation Task

It is important to note the difference between subjective attributes that occur in the explanations for decisions and the psychophysical or psychometric tasks where participants estimate the magnitude of a single attribute. The frequency of an attribute in explanations is not directly associated with its magnitude because its occurrence is related to its importance in the quality estimation and to the magnitudes of other attributes. One factor is also accessibility of an attribute (Kahneman, 2003); some attributes are more familiar and more often associated with quality, so participants may be biased toward these attributes. As clearly shown by the results of Experiment 1, participants predominantly seek one plausible reason to justify their choice and therefore use the most salient attribute in explanations, potentially masking the magnitudes of other attributes.

Similarly, when people are free to use any vocabulary that they desire, there is a possibility that attributes will not have the same meanings between participants. Some aspects of quality may also be difficult to verbalize, leading participants to use less specific expressions such as “good colors” or “good lighting.” In addition, with small, near-threshold differences, it may be difficult for naïve participants to distinguish between sharpness, graininess, or contrast.

In Experiment 2, we used the same materials and a similar method, but the choices were no longer explained; instead, the participants were asked to estimate the quality after the choices using content-specific attributes with buttons similar to those used for indicating their choices. For instance, participants were asked whether image A or B was sharper, more natural, or had better skin tone, depending on the content. These attributes were derived from the qualitative analysis of Experiment 1, representing the most important aspects of IQ in each content.

Experiment 2 had a dual purpose. First, we wanted to understand the relation between the visual magnitude of each attribute, determined by a threshold task, and the frequency of its counterpart in subjective explanations. Second, attributes clearly differ in their accuracy; we therefore wanted to explore whether certain attributes are more ambiguous than others, i.e., their meaning differs between participants, and whether the differences in accuracy are caused by “false positives,” i.e., cases where some participants have detected differences where none exist.

### Methods

#### Participants and Stimuli

The participants (*N* = 32) were sampled from the same pool as in Experiment 1, but we excluded those who had already participated in Experiment 1. They were also screened using the same vision tests. All participants passed the tests. The mean age of the participants was 27.5 years (*SD* = 4.7). Of participants, 18 were females, 13 males, and one other. The same images and contents are used as in Experiment 1.

#### Attributes in the Threshold Task

On the basis of the qualitative analysis of Experiment 1 data, we created attribute dimensions by combining opposites, e.g., “sharp” and “unsharp,” into a single dimension (for the example, “sharpness”). We then cross-tabulated these attribute dimensions with image contents and performed a hierarchical cluster analysis on the resulting table by using Chi-squared distance measure and between-groups linkage. From the resulting cluster tree (dendrogram), we selected a five-cluster solution, as a larger number of clusters would have resulted in clusters with only one content. The content-specific attribute dimensions were then selected from the most frequent attribute dimensions in each cluster. We left out uninformative and redundant dimensions, such as “good colors,” in favor of more informative dimensions, such as “color distortion” or “brightness of colors” (Table 3). The purpose was not to create an exhaustive list of attributes of each content, but to choose attributes that in our view best explained the preferences in each content.

**Table 3:** Attributes selected to Experiment 2 in each content cluster, derived from Experiment 1.

Cluster	Number of contents	Attributes				
1	5	Sharpness	Yellowishness	Appearance of skin	Color distortion	Warmth
2	10	Sharpness	Lightness	Warmth	Brightness of colors	Yellowishness
3	9	Sharpness	Exposure	Clarity	Lightness	Warmth
4	6	Sharpness	Warmth	Clarity	Graininess	Yellowishness
5	2	Exposure	Sharpness	Lightness	Naturalness	Color distortion

#### Procedure

The procedure in Experiment 2 is the same as in Experiment 1, except that the free explanations were replaced with buttons for indicating pre-selected attributes. These are similar to the buttons for indicating the preference in Experiment 1, and the participant is required to make a choice for every attribute. For example, after making the choice of the better image, the participant was asked which of the images is sharper, warmer, clearer, grainier, or more yellowish. The attribute definitions were given to participants after the instruction. The definitions were based on the attribute descriptions from the qualitative analysis of Experiment 1 data.

#### Data Analysis

Quantitative analysis followed the same approach as in Experiment 1, this time also for attribute data. In other words, we transformed the attribute estimations from choice probabilities into JND values using the logit transform.

### Results and Discussion

#### Comparison of Data of Experiments 1 and 2

We wanted to compare the associations between the probabilities of subjective attributes mentioned as a reason for choice and a more traditional psychophysics-based evaluation of that subjective attribute. Following the tradition obtained from psychophysics, we linearized the Experiment 2 data using the transformation described in Equations 1, 2. Experiment 1 attribute data are described as probabilities. While Experiment 2 data represent visibility of attributes, Experiment 1 provides second-order data of how visible attributes are subsequently used in choices, and thus, their influence on overall quality judgments. If the probability of use of an attribute is a monotonic function of its visibility, then the attribute is primary to other attributes in its importance because it does not depend on the visibility of the other attributes. It also reveals that the meaning of the attribute is clear and shared between participants.

**Figure 8:**
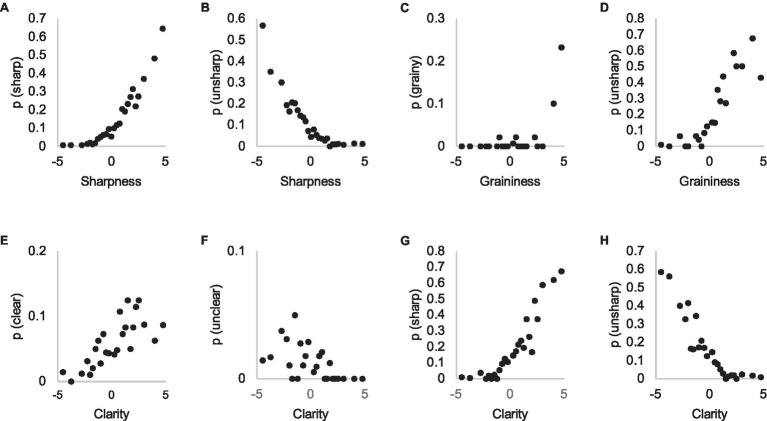
Probability of using subjective attributes ‘sharp’ **(A)**, ‘unsharp’ **(B)**, ‘grainy’ **(C)**, ‘clear’ **(E)**, and ‘unclear’ **(F)** in Experiment 1 explanations (y-axis) as a function of their visibility in threshold estimation task (x-axis, Experiment 2). In panels **(D,G,H)** different attributes are plotted in *x* and *y*-axes.

Figures 8A–D shows these probabilities for subjective attributes that were typically in pairs where differences were large. We can see very different distributions; participants mentioned the attribute ‘sharp’ quite frequently in pairs where the difference in threshold evaluation is zero JNDs, or even negative, and the relation is quite monotonic (Figures 8A,B). It appears that participants do not only notice sharpness differences easily when making IQ estimations, but actively seek them, occasionally making false detections. By contrast, “grainy” was mentioned only in pairs where the difference exceeds three JNDs (Figure 8C). When the attribute “unsharp” is plotted against the graininess dimension, we see that occurrence of the “unsharp” attribute increases when graininess is evaluated to be more than zero JNDs (Figure 8D).

The result suggests that any quality artifact that reduces visibility of details appears to be interpreted as sharpness. In other words, distinguishing the type of defect that reduces IQ near threshold is difficult and this is often referred to as “unsharpness.” On the other hand, when asked to estimate graininess or sharpness of the image in a threshold task, participants may estimate any aspect that degrades the visibility of details as such because the identity of the degradation may be difficult to classify when differences approach the threshold, leading to false detections.

We assumed that clarity, referring to such attributes as “clear” and “unclear,” would somehow combine on a perceptual level the influence of all IQ features that reduce the visibility of details (Leisti et al., 2009). Figures 8E–H show that “sharp,” instead, in many cases functions as such a general, higher level attribute, despite its usual definition as resolution. When asked to estimate clarity in Experiment 2, people have referred to the property of images that manifests as the attribute of sharpness, as it is used in explanations of Experiment 1. When differences between images are well above the threshold, participants are able to identify the attributes correctly, leading to a steep increase in their occurrence, as in the case of graininess (Figure 8C).

#### Colors

Generally, colors can be described by referring to the three dimensions of hue, saturation, and lightness. With respect to photographic images, colors can also be evaluated for their naturalness, or color distortion, defined as ΔE, which describes the color shift from original colors. Subjective color attributes, on the other hand, represent a heterogeneous and ambiguous set of descriptions of color. For instance, bright colors might refer to either saturated colors or high contrast, whereas dark colors might refer to either saturated colors or low lightness. It is also probable that participants economically use attributes that refer to more than one color dimension. On the other hand, colors are often evaluated in reference to a certain naturalness or esthetic, which is dependent on personal preferences. In Experiment 2, colors were evaluated using the four dimensions of color brightness, color distortion, warmth, and yellowishness, depending on the content.

**Figure 9:**
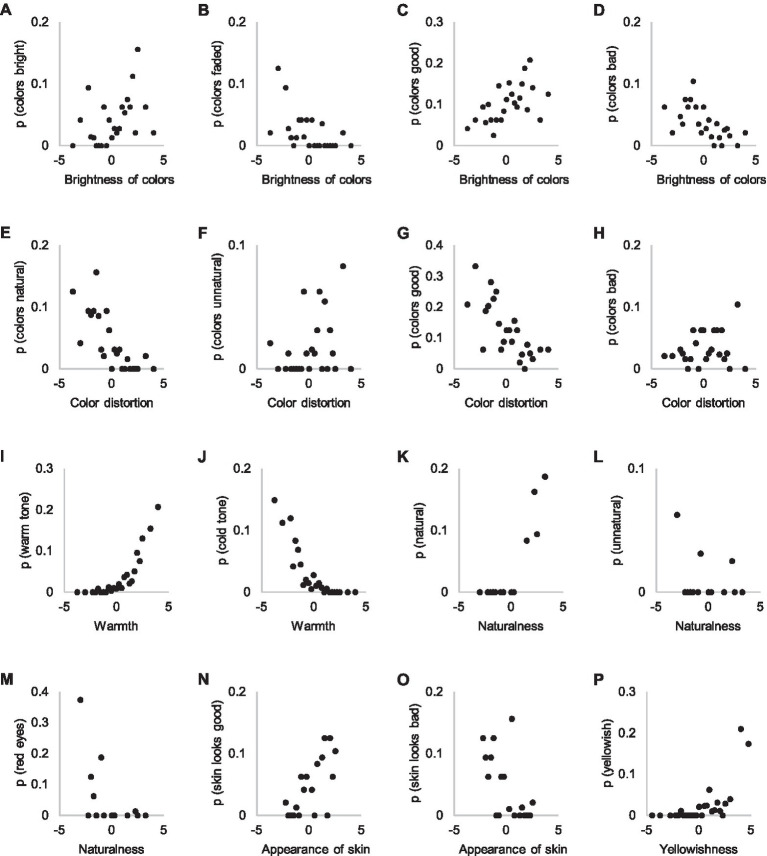
Attributes referring to bright **(A)**, faded **(B)**, good **(C,G)**, bad **(D,H)**, natural **(E)** and unnatural colors **(F)**, as well as warm tone **(I)**, cold tone **(J)**, general naturalness **(K,L)**, red eyes **(M)**, appearance of skin **(N,O)** and yellowishness **(P)** in Experiments 1 and 2. The y-axis shows the probability of an attribute in Experiment 1 explanations and the x-axis the visibility of the attribute in Experiment 2.

When the probability of using the attribute “bright colors” in Experiment 1 is plotted against the brightness of the colors dimension acquired from the threshold estimation task in Experiment 2, poor correspondence is evident (Figures 9A–D). The probable reason is that people use brightness of colors as a reason for choice only when no clear defects exist in the images. Experiment 1 data suggest that people are relatively accurate when using this attribute. Naturalness of colors suffers from a similar poor correlation between Experiment 1 and Experiment 2 data (Figures 9E,F), and the probable reason is the same as the reason concerning the brightness of colors. The subjective attributes “good colors” and “bad colors” in Experiment 1 appear to be associated with both brightness and naturalness of colors, as would be expected (Figures 9C,D,G,H).

Figures 9I,J illustrate the correlations between the warmth ratings in Experiment 2 and the probabilities of subjective attributes in Experiment 1. The consistent relation between the occurrence of “warm” and “cold” attributes in explanations and warmth estimations is striking, considering the significantly lower association with other color attributes. Warm or cold colorcast is visible over the entire image so it might not be dependent on participants’ focused attention, resulting in less varied distribution.

Participants used the subjective attribute “natural” (Figures 9K–M) in only a few pairs in Experiment 1, in which the other image was evaluated as extremely unnatural, probably due to “red eye” (Figure 9M). Reference to appearance of skin as a reason in Experiment 1 and its estimation in Experiment 2 shows a similar correspondence as the case concerning naturalness of colors; skin appearance is used as a reason only if no visible defects exist (Figures 9N,O). The attribute “yellowishness” is used quite consistently (Figure 9P). It, however, attracts attention as a reason only at more extreme levels.

#### Lightness

**Figure 10:**
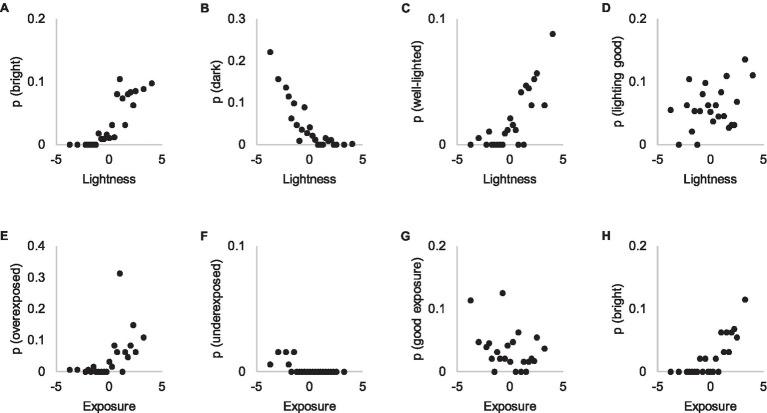
Attributes referring to brightness **(A,H)**, darkness **(B)**, lightness **(C)**, quality of lightness **(D)**, overexposure **(E)**, underexposure **(F)** and quality of exposure **(G)** in Experiments 1 and 2. The *y*-axis shows the probability of an attribute in Experiment 1 explanations and the *x*-axis the visibility of the attribute in Experiment 2.

Subjective attributes concerning lightness levels (“bright” and “dark”) are consistent with the threshold estimation task concerning lightness of the photographs (Figures 10A,B). This might be explained similarly as the consistency of using the attributes “warm” and “cold” as reasons; lightness level is widely visible in the image, requiring no voluntary attention to be noticed. In other words, perceiving lightness differences emerges from bottom-up processes that require no deliberate search. “Well-lit” corresponds well to the lighting level (Figure 10C), but “good lighting” does not (Figure 10D).

We also asked participants in Experiment 2 to estimate exposure because overexposure was a problem in certain contents. However, participants appear to be more acquainted with the concept of lightness than exposure (Figures 10E–H), which aligns quite poorly with the occurrence of subjective attributes in Experiment 1. It appears that participants do not conceptualize exposure as a continuum, instead referring to it only when certain parts of the photograph are overexposed.

#### Summary of the Results

Our task differed from typical tasks in psychophysics, which measure thresholds for individual participants. We accumulated data over a larger number of participants such that the threshold instead describes a threshold for the general population to notice a certain attribute and not an actual psychophysical threshold. Therefore, the task measures the saliency of the attribute. The use of the attribute as a reason, on the other hand, not only depends on its saliency, but also its importance, which is based on the presence of other attributes and their subjective importance.

The results suggest four different types of associations between subjective attributes used as a reason for a choice in a paired comparison task and subjective attributes as estimated in a threshold task. In the first type of association between subjective attributes as a reason and their estimation in a threshold task, the relation is unambiguous, monotonic, and sigmoidal. Attributes that belong to this type are related to sharpness, color temperature, and lightness level of the image. These image features probably capture participants’ attention easily and are visible over all images. Participants may also actively seek these attributes, so they are mentioned always when they are detected.

In the second type, the relation is highly exponential. For example, graininess is referred to only in pairs where it is clearly visible. A lower level of noise is probably interpreted as blur, is not noticed at all, or does not distract the participants. Yellowishness is referred to similarly only when it is clearly visible and interpreted as a defect; at lower levels, yellowishness appears to be associated with warmth and is not considered distracting. It therefore appears that attributes of this type are not actively sought to be used as a reason but can nevertheless be crucial when they are sufficiently salient. In other words, the first type of reasons may be related to more top-down controlled, cognitive strategy where reasons are actively sought, whereas this second type is related to saliency, therefore being a bottom-up, perceptual strategy.

The third type of association between reasons and thresholds appears linear and variable. For example, the probability that natural colors are mentioned as a reason is somewhat linearly associated with the scale values of the threshold task, but the relation is rather inconsistent. “Natural colors” have been mentioned as a reason for selecting an image even when this image is estimated to have more distorted colors than the rejected image. A similar attribute is clarity, which illustrates the possible reason for this inconsistency: when people are asked to estimate “clarity,” they are not using their own vocabulary; instead, they estimate something that they also call “sharpness” (Figure 8G). The fourth type could be described as a no-correlation type. For example, “good exposure” appears not to be related to exposure level estimations at all. These two latter types of attributes may also indicate *post hoc* rationalizations for choices that are difficult to explain.

## Experiment 3

In Experiment 1, we showed that additional time does not mean that participants report more attributes, suggesting that participants rely on a strategy that seeks one reason to justify their choice. Our examination of the heuristic decision-making in IQ estimation in previous experiments was mainly based on decision times, combined with participants’ subjective reports about the attributes that determined their choices. The IQ differences were not experimentally controlled, however, and therefore we do not know the causal relation between subjective reasons and the choices. Additionally, we do not know whether the participants are really using the strategy that the written explanations suggest.

Therefore, we analyzed decision time data from our previous experiment (Leisti and Häkkinen, 2018; referred to as Experiment 3 further in the text), where we controlled the IQ differences between the stimulus images. We either added blur or noise to the images or changed the color balance or lightness level of the images. We analyzed relations between decision times and objective differences, not subjective differences. This allows us to examine how multidimensionality of IQ affects decision times and strategies.

### Methods

#### Data

We used decision time data from a previous experiment (Leisti and Häkkinen, 2018; Experiment 1). In the experiment, participants were asked to make pairwise choices between two versions of the same image content. Because the original purpose of Experiment 3 was not to measure decision times, the precision of the data is 1 s. From this data, we included in the analyses only the condition for which the reasons for choices were given retrospectively, after each choice, because the decision times in the before condition included the time for writing the explanations. This condition had 50 participants (39 females and 11 males) with a mean age of 25.5 years (*SD* = 4.9).

There were two image contents, with a resolution of 1,920 × 1,200 pixels, and the images had been manipulated according to four different IQ parameters: blur, noise, lightness level, and color temperature. The effect of degradation of each manipulation was approximately one JND, based on pilot tests. For blur, this meant adding Gaussian blur with 0.45 SDs; for noise, adding noise with variance of 0.001 (first content) or 0.0006 (second content). We either added lightness by increasing L* channel in the L*ch color space by a value of 8 (first content) or decreasing it by a value of 12 (second content). We changed color temperature from 5,600 to 6,500 K (first content) or from 3,400 to 2,700 K. To shorten the experiment, we used only versions of images with a maximum of two manipulations at a time, thus, 11 versions of each image and 55 image pairs altogether for both image contents. The stimulus images are available from the corresponding author upon request.

Participants were asked to choose the preferred alternative of two versions of an image, presented simultaneously on two 24.1 in Eizo ColorEdge CG241W displays. Two choices were indicated using a mouse and a button on a third display. Participants went through all of the image pairs of one content before proceeding to the other and explained their choices on one content. The order of the contents only and the contents for which explanations were given was randomized and counter-balanced between the participants.

#### Analysis of the Decision Strategies

We analyzed only data that concerned pairs in which differences existed in the two most important attributes. We followed the approach developed by Glöckner and Betsch (2008) and examined decision times in cases that can divided into the four patterns presented in Table 4. In all patterns, no difference existed in the third and fourth most important attributes. In the first pattern, the two most important attributes supported the choice of alternative A. In the second pattern, a difference existed only in the most important attribute. In the third pattern, the two most important attributes contradicted each other. In the fourth pattern, a difference existed only in the second most important attribute.

**Table 4:** Attribute patterns used in Experiment 3.

	Pattern 1		Pattern 2		Pattern 3		Pattern 4	
Alternative	A	B	A	B	A	B	A	B
Most important attribute	+	−	+	−	+	−	0	0
2^nd^ most important attribute	+	−	0	0	−	+	+	−

The importance of the attribute was calculated participant-wise, based on the participants’ pairwise choices. Plus sign denotes manipulation of the attribute, minus sign denotes lack of manipulation of the attribute, and zero denotes that no difference exists between alternatives. In the last case, both images may be manipulated or not.

If the participants are applying the heuristic approach that determines the choice using only the most important available attribute, the decision times should not differ in patterns one to three and should be significantly longer in pattern 4. This is because the participants first look for the most important attribute and then proceed to the second most important attribute if differences are not found. If the participants go through all information and do not use any heuristic, the decision times should be the same in all patterns. A bottom-up, saliency-driven decision strategy would show the longest decision time in pattern 3 because attributes compete for participants’ attention and the fastest decision time in pattern 1 because both attributes draw participants’ attention in the same direction.

### Results and Discussion

Each choice in selected patterns took on average 9.3 s (*SD* = 6.4). When explanations were required, the mean decision time was 10.9 s (*SD* = 7.3) and when not, 7.6 s (*SD* = 4.7). To test the statistical significance of the decision time differences between the patterns, we performed mixed ANOVA on the log-transformed decision times, with pattern and explanations as within-participant variables, and the content and the order of the explanations as between-participant variables. ANOVA, or analysis of variance, tests the differences between means in different experimental conditions (Howell, 1997; Olejnik and Algina, 2003). We used Log transform to normalize the skewed decision time distributions.

**Figure 11:**
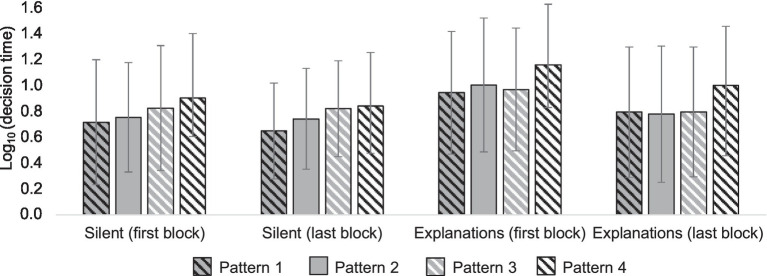
Log-transformed decision times in four patterns in different conditions (silent vs. explanations). The attribute patterns 1, 2, 3, and 4 are presented in numerical order from left to right within each condition.

Mean decision times differed between patterns [*F*(3,138) = 19.62; *p* < 0.001; partial η^2^ = 0.30], confirming our hypothesis about the heuristic nature of the decision process. Results concerning what specific heuristic, top-down or bottom-up, the participants used were mixed; whether or not the decisions were explained influenced the decision times in different patterns [*F*(3,138) = 5.04; *p* = 0.003; partial η^2^ = 0.10; Greenhouse–Geisser corrected; Figure 11], suggesting different IQ estimation strategies in different explanation conditions. When choices were explained, the decision times were the same in the first three patterns (all *p*’s > 0.5, paired *t*-test), and the decision times in the fourth pattern differed significantly from the other patterns (*p*’s < 0.001, paired *t*-test). In the silent condition, decision times in all patterns differed, except between patterns 2 and 4. Content did not have an effect on the decision times, nor did it interact with any other variable. Order of the explanations condition interacted with the explanations condition, meaning that decision times were shorter in the last block [*F*(1,46) = 15.87; *p* < 0.001; partial η^2^ = 0.26].

#### Does Explaining Affect Decision Heuristics?

The results suggest that in conditions where explicit reasons for choice are required the participants use a more top-down controlled approach, in which they go through attributes in their order of importance and choose the alternative that is better according to that attribute. In the silent condition, participants use a more bottom-up oriented approach, where attributes appear to compete for attention, causing a delay when the attributes contradict each other and facilitating the decision when the attributes are in unison.

Experiment 3 differed from two previous experiments in the sense that quality degradations always had the same magnitude and were drawn from the set of four different degradations. The participants also made a large number of choices within content with same quality degradations. Participants learned the possible differences between the images and actively sought them. This might have influenced the strategy because the participants generally knew what to expect from the differences between the images. These expectations might have further facilitated a more top-down oriented strategy. Nevertheless, participants appear to employ a heuristic, one-reason decision strategy in both cases.

#### Top-Down vs. Bottom-Up Strategies in IQ Estimation

Experiment 3 illustrates how cognitive top-down strategies differ from bottom-up perceptual strategies. The top-down approach is evoked not only when participants must justify their estimations, but also when they have more expertise or experience in the task or they are instructed to do the task a certain way, for instance, by attending to certain key attributes. A top-down strategy, in other words, requires prior knowledge, which facilitates an information search for attributes that are more diagnostic in the task. The top-down strategy appears to yield more consistent results (Leisti and Häkkinen, 2016), but may not be the prevalent approach in IQ estimation among end users who do not have established strategies to approach quality.

## General Discussion

The purpose of this study was to investigate the cognitive basis of subjective visual quality estimation by examining how physical quality differences between images manifest in experience as subjective quality attributes and how these attributes are exploited in decision-making in a 2AFC quality assessment task. The point of departure here was the IBQ method (Radun et al., 2008, 2010), which we applied in Experiment 1. According to the IBQ approach, any rating of subjective IQ is a result of a subjective experience of quality-related features, their interpretation, and their role in the accompanying decision-making (Radun et al., 2008, 2010; Nyman et al., 2010). In Experiment 2, we further investigated how the use of the attributes in Experiment 1 is related to their visibility in the 2AFC threshold task, operationalized as JND values. In Experiment 3, we examined the participants’ heuristics by examining the decision times in different attribute configurations.

The general finding of this research was that subjective IQ estimation is, above all, a heuristic mental activity. Participants’ choices appear to stem from a strategy in which they try to find a reason that justifies the selection of one alternative and the rejection of the other. Not only is this evident from their reported reasons for choices, also the response time analysis shows that participants use most of their mental effort to find a single attribute; additional decision time does not materialize in a larger set of attributes, which would suggest a more compensatory strategy. The participants only aim to find a single reason for selecting an alternative that both differentiates the alternatives and has some sort of valence. They appear to avoid attributes that are not justifiable due to their low valence or accuracy. When participants have found a salient difference with clear valence, they make a fast choice. This leads to clear overall quality difference when data accumulate for all participants. However, when the overall quality difference is small, participants are unable to immediately find such a salient reason for their choice. This may result from small overall differences between alternatives, conflict between attributes, or attributes that are preferential in nature.

One-reason decision-making in the task probably stems from the fact that many IQ attributes, like noise, blur, and contrast, are separable, therefore requiring divided attention. As the IQ assessment is a relatively tedious and repetitive task and divided attention toward different attributes increases mental effort, the participants adapt rapidly to a less demanding strategy based on the most important attribute (Leisti and Häkkinen, 2018). This may be one way that learning and subsequent expertise diminish the cognitive effort in judgment and decision-making tasks (Garcia-Retamero and Dhami, 2009), leading to more efficient processing.

Interestingly, individual participants appear to have used the one-reason strategy even when differences were small, which was contrary to our expectations. We anticipated that small differences would require participants to present additional evidence to support their choices. However, we found that participants’ attributes diverged when quality difference was around two JNDs. The amount of conflict, operationalized as number of conflicting attributes in decision space, drops drastically after this limit, as does decision times. At the same time, accuracy of the attributes increases. However, even if quality differences are under two JNDs, they are not necessarily meaningless; images can still be visibly different and have failed in different ways such that the optimal image would be a compromise between the two. Therefore, deeper understanding about the reasons for choices would be useful.

### Visibility of Attributes and Their Occurrence in the Subjective Decision Space

Experiment 2 shows that occurrence of attributes in subjective explanations is not monotonically related to their visibility, as defined in the threshold task. There are multiple reasons for this. First, the subjective attributes are used as reasons for choice; thus, if the attribute does not appear relevant for decision-making, it is not mentioned due to a heuristic strategy, even if it is clearly visible. This is likely caused by some other more salient attribute. This “masking” phenomenon is a probable reason for the Minkowski-type of summation of the quality degradation of separate quality defects (e.g., Engeldrum, 2002; Keelan, 2002; Jin et al., 2017).

The second reason is that when the IQ difference is below two JNDs, participants may have difficulties in attributing the differences to specific attributes. Instead, they are likely to refer generally to “sharpness.” Graininess or yellowishness, for example, are mentioned only if they are well above the threshold level, leading to a highly exponential function between visibility and counts in Experiment 1.

The third reason might lie in the way that participants interpret the task, which may influence the differences that they seek from the images. Asking research participants to evaluate IQ may induce some participants to seek certain attributes that they think are relevant for quality evaluation. These attributes may result from typical narratives that people use to describe the quality of cameras, displays, and other imaging devices. One such attribute is evidently sharpness, and people appear to interpret any lack of detail as unsharpness, even if it is caused by lack of contrast or noise.

Although the choices concerning cases where quality differences are small appear somewhat random, the focus on subjective attributes makes them informative. From the more variable individual choices and explanations, a more general picture converges when both choices and attribute data accumulate, describing a decision space that unfolds in each pair. Although this decision space is more variable when quality differences are small, it is simultaneously more informative by providing more subjective attributes than the cases where participants are unanimous in their estimations. Attribute data can also inform regarding whether the reason for more equal choice distribution in a pair results from similarity or from large differences that cancel each other out. Images can, for instance, have a very small difference in sharpness or they can have large differences in both sharpness and noise, but because one image is noisy and the other is blurry, the participants have difficulty identifying the better image.

A traditional approach toward IQ, relying on psychophysics, has been criticized due to its over-emphasis on thresholds because knowledge about thresholds does not offer understanding about the use of supra-threshold information in subjective quality estimation. The solution offered by the IBQ approach suggests that supra-threshold information is interpreted from multiple subjective perspectives of research participants, forming a decision space from which the heuristic reasons for quality decisions are sought (Nyman et al., 2010). This study further clarifies this process by suggesting that people need only one reason for selecting a better image. Future research should focus on the factors that determine these reasons in different contexts. Earlier studies suggest that attentional processes are important, both bottom-up controlled processes that are related to visual saliency and top-down processes that are based on semantics and task interpretations (Radun et al., 2014, 2016).

### Are Reasons Given in Explanations the Real Reasons for the Choice?

Would participants rely on a less heuristic strategy if they were not required to give reasons for their choices? In other words, do the experimental protocols cause the apparent reliance on one-reason decision-making in the task, and would the participant use a more compensatory approach for their choices if not required to explain them? We have studied this elsewhere in several experiments (Leisti et al., 2014; Leisti and Häkkinen, 2018), and the answer seems to be no. On the contrary, explaining appears to *increase* attention to less important attributes, whereas silent deciding results in more emphasis on the most important attribute.

In addition, we want to clarify the nature of the subjective attribute data used in this study. It should not be seen as process data, like the data derived from the analysis of thinking-aloud protocols (Ericsson and Simon, 1980; Ericsson and Fox, 2011), but subjective verbal description of experiences that a group of participants regard as significant in their judgments and choices. This subjective data should be approached from a general level, as distributions accumulated over several participants, similarly to the choice distributions (Nyman et al., 2010). It reveals very little about the actual decision-making processes of a single individual, instead describing the potential decision space that can opens up to them and from which the attributes of the choice can be sought. For instance, our results suggest that the set of experiences that participants consider relevant in their quality judgments is much more variable when differences between alternatives are small rather than large. Variation in reported reasons for choices co-occurs with variation of choices, supporting the validity of the verbal data.

The difference between process data, provided by concurrent thinking-aloud protocols (Ericsson and Simon, 1980), and the IBQ method is what the attributes are assumed to be. Whereas the concurrent thinking-aloud protocols are supposed to study the actual process of judgment and decision-making, the IBQ approach examines the attributes that participants consider relevant in their judgments. We thus conceptualize explaining as a metacognitive task; it is a form of monitoring performed on the subjective experiences and associated preferences and the subsequent verbalization of the beliefs that have emerged from this monitoring (Leisti and Häkkinen, 2016). In this way, it resembles the sensory evaluation methods in the evaluation of food and beverages (Varela and Ares, 2012) or audio quality (Lokki et al., 2012). Our framework therefore aims to bridge the gap that currently exists between sensory evaluation studies and micro-economic research on consumer choices.

### General IQ Estimation Heuristic

Following the fast-and-frugal heuristics tradition, our data give some indications about the possible heuristic decision tree used by the participants. First, participants appear to reject the image that is clearly failed due to misfocus, over-exposure, noise, or some other salient weakness. Users might have learned this in their everyday use of cameras. If this does not give a clear result, participants seek other salient differences, for example, in the visibility of details—or in “sharpness” in their own words. Visibility may, however, be degraded not only by blur, but also by noise or low contrast. From the bottom-up perspective, visual saliency appears to have a significant role in heuristics; if there is a clear quality attribute that captures viewers’ attention, it usually is used as a heuristic reason for choice. If no salient difference captures viewers’ attention, viewers allocate more effort to the task and seek minor differences in a top-down manner, giving more emphasis to artifactual and less emphasis to preferential attributes. If no differences are found in this respect, any difference suffices as a reason, and sometimes an *ad hoc* meaning is generated for the difference to justify its role in the task.

Our results have possible implications also for objective IQ metrics. Instead of predicting directly the participants’ mean quality ratings, objective metrics could predict choices and simulate the decision tree that primarily uses bottom-up information emerging in the decision space. The MOS values could then be calculated from these simulated choice distributions using appropriate scaling techniques. Predicting a choice should be significantly simpler that predicting the mean values of ratings accumulated over a large number of viewers. In addition, in choices, the underlying heuristic estimation process becomes explicit, unlike in the MOS, where the individual processes can be anything.

## Conclusion

In IQ estimation, psychophysics and visual thresholds for defects have played significant roles; an important question is what happens when visual features exceed the thresholds. Knowledge about the human visual system cannot predict the meaning of visible information and how it is used when judgments about quality are required. This study aimed to answer this question by analyzing the choices and subjective explanations given for the choices.

We found that the general strategy of individual participants stays the same independent of quality level and image content; the choice and the rejection can usually be explained by referring to a single subjective attribute. Differences between different quality levels manifest in the number of different attributes, i.e., the decision space (Nyman et al., 2010), which unfolds to participants. From this space, the most subjectively salient feature acts as a reason for choice in individual participants. A large quality difference is associated with a single salient feature, toward which the participants’ attention converges, leading to a unanimous choice distribution. Lack of such salient quality feature causes attention to diverge to several attributes, resulting in variation in choice distribution. This also forces participants to rely either on attributes whose overall meaning to the quality is more ambiguous or on near-threshold attributes, leading to less accurate detection. This dilutes the overall quality difference.

Although this research concerned decisions related to visual quality estimation, we see no reason why a similar framework would not be relevant in any case of multi-attribute decision-making. Firstly, one should understand the decision space, which describes the alternatives and their attributes from the decision-makers’ subjective—not the experimenter’s “objective”—viewpoint. Secondly, there should be understanding of how decision-makers adopt a set of attributes for reasons for their choices from this space.

Our results support the now widely accepted idea that people often make decisions using one heuristic reason only (Gigerenzer et al., 1999). It is also evident that we must shed light on the set of reasons from which the chosen reason is selected and the basis for the selection. This experiment suggests that the reason applied is usually the one that is first visible to the participant. The prevailing heuristic, therefore, appears to rely on saliency of the attributes, or accessibility in Kahneman’s terms (Kahneman, 2003). Still, we have significant problems understanding the idiosyncratic processes that determine the identities of the attributes in the decision space in the first place. This issue goes back to subjective experience, or phenomenal consciousness, and the factors that determine its contents (Morsella, 2005).

## Data Availability Statement

The raw data supporting the conclusions of this article will be made available by the authors, without undue reservation.

## Ethics Statement

The studies involving human participants were reviewed and approved by the Ethics Review Board in Humanities and Social and Behavioral Sciences of the University of Helsinki (decision no. 40/2017). The patients/participants provided their written informed consent to participate in this study. Written informed consent was obtained from the individual(s) for the publication of any potentially identifiable images or data included in this article.

## Author Contributions

TL wrote the original draft, created the experiments, and performed the statistical analyses. TL, J-LO, MV, V-TP, and JH conceptualized and designed the experiment. J-LO and MV generated the stimuli for the first two experiments. TL generated the stimuli for the third experiment. JH contributed to the drafting and critical revision of the article, acquired the funding, and supervised the project. All authors contributed to the article and approved the submitted version.

## Funding

This study received funding from Huawei Technologies Oy (Finland). The funder had the following involvement with the study: three employees of the funder (J-LO, MV, and V-TP) were responsible for generating the stimuli and participated in designing the experiments 1 and 2. Open access publishing was funded by University of Helsinki Library.

## Conflict of Interest

MV, J-LO, and V-TP were employed by Huawei Technologies Oy (Finland) Co., Ltd.

The remaining authors declare that the research was conducted in the absence of any commercial or financial relationships that could be construed as a potential conflict of interest.

## Publisher’s Note

All claims expressed in this article are solely those of the authors and do not necessarily represent those of their affiliated organizations, or those of the publisher, the editors and the reviewers. Any product that may be evaluated in this article, or claim that may be made by its manufacturer, is not guaranteed or endorsed by the publisher.

## Footnotes

^1^We report the English translations of the original Finnish attributes, which should be taken into account when making interpretations about the meaning of the specific attributes.
