# Supplementary material for: The Fewer Reasons, the More You Like It! How Decision-Making Heuristics of Image Quality Estimation Exploit the Content of Subjective Experience
Source: Front Psychol. 2022 Jun 21;13:867874. doi: 10.3389/fpsyg.2022.867874 (PMC9253696; doi:10.3389/fpsyg.2022.867874)
Supplement: Supplementary file 1 [file Table_1.DOCX]

**Appendix A**

*All subjective attributes mentioned more than once from the qualitative analysis, with their count, number of participants using the attribute, number of contents where attribute were mentioned, and accuracy and valence of the attribute.*

| Attribute | Count | No of participants | No of contents | Accuracy | Valence |
| --- | --- | --- | --- | --- | --- |
| Sharp | 944 | 32 | 32 | 0.8 | 0.94 |
| Unsharp | 724 | 31 | 32 | 0.83 | -0.95 |
| Colors good | 596 | 31 | 32 | 0.67 | 0.98 |
| Lighting good | 338 | 29 | 32 | 0.79 | 0.99 |
| Dark | 258 | 29 | 27 | 0.97 | -0.88 |
| Clear | 246 | 29 | 32 | 0.69 | 0.84 |
| Warm tone | 209 | 27 | 31 | 0.95 | 0.77 |
| Bright | 189 | 25 | 32 | 0.96 | 0.01 |
| Overexposed | 184 | 29 | 23 | 0.96 | -0.92 |
| Cold tone | 167 | 25 | 29 | 0.95 | -0.89 |
| Colors bad | 162 | 24 | 28 | 0.77 | -0.98 |
| Yellowish | 158 | 25 | 23 | 0.99 | -0.92 |
| Natural | 148 | 24 | 31 | 0.85 | 0.97 |
| Colors natural | 127 | 27 | 30 | 0.91 | 0.95 |
| Lighting bad | 125 | 28 | 26 | 0.89 | -0.97 |
| Exposure good | 121 | 23 | 26 | 0.87 | 1 |
| Colors_bright | 105 | 23 | 21 | 0.92 | 0.68 |
| Well-lighted | 104 | 23 | 22 | 0.94 | 0.88 |
| Quality better | 95 | 21 | 28 | 0.94 | 0.87 |
| Skin looks good | 93 | 18 | 18 | 0.85 | 1 |
| Atmosphere bad | 87 | 17 | 28 | 0.91 | -0.93 |
| Colors faded | 80 | 16 | 26 | 1 | -0.98 |
| Skin looks bad | 80 | 17 | 20 | 0.9 | -1 |
| More contrast | 71 | 18 | 25 | 0.94 | 0.75 |
| Reddish | 69 | 18 | 14 | 0.91 | -0.8 |
| Colors light | 63 | 19 | 21 | 0.97 | -0.14 |
| Grainy | 62 | 15 | 10 | 1 | -0.94 |
| Unclear | 62 | 19 | 24 | 0.9 | -0.97 |
| Atmosphere good | 59 | 18 | 23 | 0.93 | 1 |
| Balanced | 56 | 13 | 20 | 0.89 | 0.96 |
| Color balance better | 50 | 14 | 20 | 0.92 | 0.6 |
| Soft | 49 | 15 | 21 | 0.96 | 0.92 |
| Gray | 44 | 10 | 16 | 1 | -1 |
| Flash irritating | 39 | 14 | 5 | 0.95 | -0.9 |
| Bad quality | 38 | 13 | 19 | 0.89 | -0.79 |
| Colors unnatural | 38 | 17 | 16 | 0.95 | -0.84 |
| Blurry background | 31 | 9 | 13 | 0.95 | 0.35 |
| Smooth | 31 | 11 | 17 | 0.94 | 0.87 |
| Less contrast | 29 | 10 | 17 | 1 | -0.38 |
| Other color distortion | 28 | 9 | 14 | 1 | -0.71 |
| Unnatural | 25 | 13 | 17 | 1 | -0.76 |
| Blurry target | 24 | 9 | 10 | 1 | -0.67 |
| Pale | 24 | 8 | 9 | 1 | -1 |
| Artefact | 18 | 11 | 8 | 1 | -0.89 |
| Red eyes | 17 | 8 | 1 | 0.88 | -0.88 |
| Not bright | 15 | 9 | 11 | 1 | 0.2 |
| Exposure bad | 12 | 5 | 8 | 1 | -0.83 |
| No artefacts | 12 | 7 | 5 | 1 | 1 |
| Hard | 10 | 6 | 8 | 1 | -1 |
| Underexposed | 10 | 3 | 5 | 1 | -0.8 |
| Colors dark | 8 | 6 | 7 | 1 | 0 |
| Flash not irritating | 8 | 4 | 4 | 1 | 1 |
